# Validation of a Machine Learning-Derived Algorithm for the Measurement of Facial First Impressions

**DOI:** 10.1007/s00266-026-05835-x

**Published:** 2026-05-26

**Authors:** Heike Klepetko, Georg Dorffner, Thomas Schulz, Maris Klepetko, Arthur Swift

**Affiliations:** 1 Plastic, Reconstructive and Aesthetic Surgeon, Radetzky Villa Private Clinic, Cobenzlgasse 46, 1190 Vienna, Austria; 2 Center for Medical Data Science, Medical University of Vienna, Vienna, Austria; 3 Deep Learning Specialist, Aemos GmbH, Vienna, Austria; 4 Plastic, Reconstructive and Aesthetic Surgeon, Swift Beauty, Montreal, Canada

**Keywords:** First impressions, Facial analysis, Aesthetic facial treatments, Artificial intelligence, Validation

## Abstract

**Background:**

Facial first impressions are formed within milliseconds and play a pivotal role in social interactions. These rapid judgments influence how individuals are perceived in terms of personality. Traditional assessments of these first impressions are subjective and prone to interobserver variability. Artificial intelligence (AI) presents a tool for standardizing and objectifying such evaluations, offering reproducibility and scalability for clinical and research settings. Since aesthetic treatments of the face might alter how a person’s face is perceived, measurability is of crucial importance.

**Objectives:**

This study validates an AI algorithm trained to predict eight facial impression traits by comparing its predictions with crowd-sourced human evaluations.

**Methods:**

A test set of a total of 1795 standardized facial images was rated by 30 independent raters per image using Amazon Mechanical Turks, evaluating eight traits: attractive, trustworthy, healthy, happy, rested, dominant, threatening, and sexy. Additionally, raters were asked how likely it is that the presented face underwent aesthetic treatment (naturalness). These ratings and AI predictions were statistically compared using Pearson´s correlation coefficient (r) and intraclass correlation coefficient (ICC).

**Results:**

Pearson´s correlation coefficient showed a strong positive linear relationship for all traits, including naturalness (*r *> 0.7). According to Cicchetti standards, the intraclass correlation coefficient (ICC) showed excellent results for the traits attractive, trustworthy, rested, happy, healthy, sexy, and naturalness. For the traits dominant and threatening, it was fair, yet still led to a highly significant correlation.

**Conclusion:**

The validated algorithm demonstrates reliability for all traits, including naturalness, offering a valuable tool for aesthetic professionals seeking objectivity in first-impression-based facial assessment and treatment planning.

**No Level Assigned:**

This journal requires that authors assign a level of evidence to each submission to which Evidence-Based Medicine rankings are applicable. This excludes Review Articles, Book Reviews, and manuscripts that concern Basic Science, Animal Studies, Cadaver Studies, and Experimental Studies. For a full description of these Evidence-Based Medicine ratings, please refer to the Table of Contents or the online Instructions to Authors www.springer.com/00266.

## Introduction

### Facial Perception and First Impressions

Facial perception plays a fundamental role in the rapid formation of first impressions. Research shows that we form impressions—such as whether a person is trustworthy or threatening—within just 100 milliseconds of seeing an unfamiliar face [1, 2]. These judgements occur automatically even before we are aware of them [3, 4] and strongly influence social interactions and decision making across various contexts.

Brain studies demonstrate that these impressions are not only formed very quickly, but involuntarily, without conscious effort [5, 6]. This rapid response likely developed as an evolutionary mechanism to help us assess potential social threats or allies almost instantaneously [3]. Theoretical models grounded in evolutionary psychology suggest, that these impressions arise from overgeneralizing facial cues that historically helped humans detect fitness, familiarity, or emotional intent [7, 8]. For example, a wide facial width-to-height ratio is associated with perceptions of dominance and aggression and may even predict leadership success in corporate settings [9]. Similarly, facial cues of attractiveness and trustworthiness can shift public perception in political elections [10–13].

The consequences of first impressions are profound. Quick judgements based solely on facial appearance can influence legal sentencing, hiring decisions, or financial trust among others [1, 8, 9, 14]. Individuals perceived as less attractive or more unusual are often judged more negatively, even when those impressions are inaccurate [8, 14].

In conclusion, it can be stated that exposure to facial features triggers intrinsic evaluations that are important not only in psychological theory but also in real-world settings by determining how we interact with each other [14].

Based on this, patients seeking aesthetic treatments are increasingly motivated by goals that go beyond physical rejuvenation. While the desire to appear more attractive remains common, many individuals pursue procedures to enhance self-confidence, reduce social anxiety, and improve professional and interpersonal perceptions [15, 16]. However, without feedback, treatments often do not target the desired goal. Minimally invasive treatments are often chosen to align facial appearance with internal identity and to avoid negative first impressions associated with fatigue, anger, or sadness [17, 18]. Importantly, motivations are often rooted in psychological well-being and a desire to influence how others perceive traits such as competence, approachability, and vitality [19]. In this context, naturalness is a critical outcome in aesthetic medicine. Natural-looking results are essential for maintaining authentic facial perception and therefore play a key role in fostering favorable social perception. [20, 21].

Aesthetic medical interventions consistently influence the way individuals are perceived by others. Across diverse treatment modalities, including Botulinum-Toxin-A, dermal fillers and surgical interventions like facelifts or brow lifts, patients are more likely to be perceived as attractive, trustworthy, competent, and emotionally positive after intervention [17, 19, 22–28]. These treatments frequently reduce impressions of sadness, anger, fatigue, or unapproachability, replacing them with perceptions of happiness and healthiness [19, 23, 26, 27, 29]; hence, aesthetic treatments can reshape how a person is perceived in daily interactions. Taken together, the literature confirms that aesthetic medicine plays a significant role in influencing immediate social judgments and impressions, reinforcing its psychosocial relevance.

Facial perception and first impressions are commonly evaluated using a combination of observer-based assessments, standardized photographs, and psychometric instruments [19–21, 25, 27, 28]. Validated instruments such as the Global Aesthetic Improvement Scale (GAIS) and Face-Q modules are also used to capture evaluations of facial outcomes, including self-perceived naturalness, emotional well-being, and social impact [16, 20]. However, these validated instruments lack specific measurements of social perception [17, 18]. Moreover, current scales lack consensus definitions or standardized thresholds for concepts such as facial perception and naturalness, which complicates cross-study comparisons [20].

### The Role of Artificial Intelligence in Measuring Social Perception

Artificial intelligence (AI) systems present several compelling advantages for measuring facial perception and first impressions, offering a transformative alternative to traditional, observer-dependent methods. First and foremost, AI provides a high level of objectivity and reproducibility. Unlike human raters, AI algorithms apply standardized criteria consistently across diverse facial inputs [30, 31]. This reduces study specific inter-rater variability and enhances the reliability of assessments related to perceived traits such as attractiveness, trustworthiness, and more.

Furthermore, AI systems based on deep learning, such as convolutional neural networks (CNNs), can process large volumes of facial images rapidly and identify subtle patterns in facial structure that correlate with human social judgements [31, 32]. These systems can quantify changes in facial perception pre- and post-intervention with granular detail – providing measurable metrics that track improvements or unintended effects following aesthetic procedures [33]. Additionally, AI allows for continuous, scalable, and real-time assessment across diverse populations when properly trained on heterogeneous datasets [34]. The scalability is particularly advantageous for integrating automated perception evaluation into clinical workflows or digital applications, offering practitioners a powerful tool for both pre-treatment analysis and outcome validation.

To ensure the accuracy, reliability, and clinical relevance of measurements related to facial perception, the validation of AI systems in aesthetic medicine is essential. As these tools potentially influence treatment planning, outcome evaluation, and patient communication, rigorous validation is critical to avoid misleading conclusions. While AI models have demonstrated high performance in predicting facial attributes such as age and attractiveness [31, 32], real-world validation remains a limiting factor for widespread clinical adoption and more complex attributes [35]. Recent literature has highlighted that the growing use of AI in medicine raises substantial ethical and regulatory concerns, particularly regarding algorithmic bias, transparency, data privacy, and insufficient validation. Systematic reviews and ethical frameworks consistently highlight that rigorous validation and explicit bias assessment are essential prerequisites for the responsible integration of AI-based tools into clinical practice [36–38].Validating AI for measuring facial perception is not only a methodological imperative but also a prerequisite for establishing clinical trust and maximizing the psychosocial value of aesthetic interventions.

Therefore, the aim of the study was to validate a machine learning algorithm designed to predict facial first-impression traits from standardized 2D, neutral, frontal images across eight perceptive traits: attractive, trustworthy, healthy, happy, rested, dominant, threatening, sexy, and how natural a face appears, using a robust human-rated dataset as a benchmark. We hypothesized that algorithmic predictions would demonstrate strong agreement with aggregated human ratings, supporting the use of AI as an objective measurement tool for social perception in aesthetic medicine.

## Materials and Methods

### AI Model Development

The artificial intelligence algorithm evaluated in this study was developed between 2022 and 2024 by the authors in collaboration with data scientists at Aemos GmbH, Austria, as a research and development project focused on perception-based facial analysis. The model is accessible on https://studies.aemos.at. The present manuscript reports its first independent validation for scientific purposes.

The training dataset consisted of 7147 frontal neutral-expression facial images obtained from prospectively collected clinical image repositories and standardized photographic datasets, for which written informed consent for scientific use was obtained. Images were collected prior to the present validation study and were not reused in the test dataset.

A convolutional neural network (CNN) based on a customized ResNet50 [39] architecture with pretrained weights was trained to predict nine facial perception traits. The model was trained using the above-mentioned 7147 frontal facial images. Each image was rated by 60 independent human evaluators using a five-point Likert-type scale. For data collection, Amazon Mechanical Turks [40] (M-Turks) were used for crowdsourcing. All M-Turk raters completed a screening questionnaire and two attention checks were embedded into the rating task. Raters who failed the two attention checks were excluded from the dataset.

Traits assessed included attractiveness, trustworthiness, healthiness, happiness, rested appearance, dominance threatening appearance, sexiness, and perceived likelihood of prior aesthetic treatment (naturalness).

The final layer was configured with one output node per trait, each using a sigmoid activation function. Ground truth trait scores were scaled to the [0, 1] range to match the network’s output. The model was optimized using mean squared error (MSE) loss. Preprocessing included face cropping to ensure consistent facial framing, followed by resizing and pixel value normalization. To improve generalization, both image-level and feature-level augmentations were applied. Robustness was addressed through these augmentations and formally measured as a metric, reflecting the model’s prediction stability across images with small input variations.

Demographic distributions of raters and images among the dataset for training of the CNN were recorded to assess representativeness (Table 1 (raters) and Table 2 (images)).

**Table 1:** Demographics among raters (training set)

Ethnicity		Age		Gender	
White	57.7%	18–29 yrs.	28.9%	Female	39.7%
Black	17%	30–39 yrs.	32.9%	Male	59.9%
Asian	11.2%	40–49 yrs.	24%	Others	0.3%
Latino	10.4%	50–59 yrs.	11.3%		
Others	3.7%	> 60 yrs.	2.9%

**Table 2:** Demographics among images (training set)

Ethnicity		Age		Gender	
White	53.8%	18–34 yrs.	54.4%	Female	51.3%
Black	28.5%	35–55 yrs.	42.6%	Male	48.7%
Asian	9%	> 55 yrs.	2.9%	Others	0%
Latino	7.5%				
Others	1.1%				

### Validation of the Developed Algorithm

To validate the AI model, its predictions were compared to human ratings of a test set of images. Validation was performed on a fully independent dataset that was not used during model development. The test dataset comprised 1795 frontal, standardized, neutral-expression facial images collected from databases of aesthetic clinics. For all images written informed consent was given. Each image was evaluated by 30 unique human raters via M-Turk in the same manner than applied to the dataset for training, ensuring statistical independence between training and validation phases.

The demographics of the test set is shown in Table 3 (raters) and Table 4 (images).

**Table 3:** Demographics among raters (test set)

Ethnicity		Age		Gender	
White	57.7%	18–29 yrs.	28.9%	Female	39.6%
Black	17%	30–39 yrs.	32.9%	Male	60%
Asian	11.2%	40–49 yrs.	24%	Others	0.4%
Latino	10.4%	50–59 yrs.	11.3%		
Others	3.7%	> 60 yrs.	2.9%

**Table 4:** Demographics among images (test set)

Ethnicity		Age		Gender	
White	54.8%	18–34 yrs.	55.9%	Female	51.5%
Black	27.5%	35–55 yrs.	41.4%	Male	48.5%
Asian	9.5%	> 55 yrs.	2.6%	Others	0%
Latino	6.9%				
Others	1.1%				

### Robustness

The robustness score evaluates the model’s stability when subjected to small perturbations in its input. By calculating the standard deviation of predictions for slightly altered versions of the same image, the metric reflects how consistent the model’s predictions are under minor input changes. Image permutations involve spatial transformations, such as translation (±2% of the image width or height) and rotation (± 2°), as well as photometric modifications, including variations in brightness, saturation, contrast, and hue. A smaller value indicates greater robustness, as the model’s predictions remain relatively stable despite variations in the input.

The robustness scores of our AI model are 4.7% for attractiveness, 6.7% for trustworthiness, 6.0% for healthiness, 7.1% for happiness, 6.7% for awake, 8.4% for dominant, 7.4% for threatening, and 5.4% for sexy.

### Bias

Addressing fairness and systemic bias in artificial intelligence systems is essential to prevent the reinforcement of existing societal inequalities and to support ethical and equitable outcomes. As outlined in the NIST [41] framework, bias in AI can arise from multiple sources, including imbalances in training data, annotator-related effects, and broader systemic or societal structures embedded within organizational practices. Such biases may be reflected in both datasets and algorithmic outputs, potentially leading to differential model behavior across demographic groups. For this reason, bias assessment and mitigation must be treated as an ongoing process that extends beyond initial model development and requires continuous monitoring and evaluation.

Firstly, potential sources of bias within the training data were examined by analyzing the demographic distribution of both facial images and human annotators to ensure significant representation of major racial and ethnic groups.

To further assess fairness in model predictions quantitatively across ethnic groups, a fairness metric was applied during model development. This metric calculates the deviation between the mean prediction for each ethnic group and the overall mean prediction across all groups, with the averaged deviations serving as an indicator of group-level disparity. This approach enables the identification and evaluation of systematic differences in model outputs across demographic subgroups and provides a structured basis for targeted corrective measures. The formula used to calculate the fairness metric was the following:

$$\mathrm{Fairness}\;\mathrm{metric}\,\left( \% \right) = 100* \frac{{|\mu (group_{i} ) - \mu \left( {all} \right)|}}{{\sigma \left( {all} \right)}}$$

To disentangle ethnicity-related effects from gender-related influences, images were first stratified by gender prior to further analysis. Subsequently, both images and annotators were grouped by ethnicity, and a rating matrix was constructed in which each cell represents the mean rating assigned by one ethnic rater group to images of another ethnic group. Table 5 shows a rating matrix of “attractiveness” as an example.

**Table 5:** Rating matrix for “attractiveness”: differences of mean scores between individual rater group and all other groups incl. SD

Images	Raters			
	Asian	Black	Latino	White
Female	2.267 ± 0.578	2.591 ± 0.622	2.547 ± 0.628	2.5 ± 0.609
Asian F	0.062 ± 0.553	0.121 ± 0.537	0.044 ± 0.541	0.063 ± 0.52
Black F	− 0.188* ± 0.496	0.058 ± 0.593	− 0.161* ± 0.543	− 0.262* ± 0.538
Latino F	0.088 ± 0.514	0.105 ± 0.621	0.051 ± 0.604	0.034 ± 0.573
White F	0.014 ± 0.641	− 0.17* ± 0.625	− 0.012 ± 0.685	0.024 ± 0.663
Male	2.022 ± 0.428	2.234 ± 0.487	2.135 ± 0.491	2.137 ± 0.382
Asian M	− 0.063 ± 0.393	− 0.028 ± 0.478	− 0.084 ± 0.471	− 0.07 ± 0.344
Black M	− 0.014 ± 0.397	0.052 ± 0.496	0.012 ± 0.446	− 0.102* ± 0.365
Latino M	− 0.005 ± 0.43	0.1 ± 0.527	0.006 ± 0.542	− 0.008 ± 0.416
White M	0.022 ± 0.452	− 0.065* ± 0.468	0.017 ± 0.503	0.054* ± 0.381

^*^Significance: *p*_value < (005/32) according to Bonferroni

For each target group, the mean rating provided by a specific rater group was compared with the aggregated mean rating from all other rater groups. When no statistically relevant differences were identified, no adjustments were applied. However, when systematic imbalances were detected, label adjustments were introduced during data preprocessing by subtracting the identified group-specific deviation from the corresponding ratings.

These adjustments were applied exclusively during the training phase and were not imposed on the independent validation dataset. The resulting overall fairness-score of our model is 10.5%.

This approach aims to reduce the influence of systematic rater-related biases while preserving genuine perceptual variability inherent to facial impression judgments. Nevertheless, it is important to emphasize that agreement between AI predictions and human consensus ratings does not imply the elimination of bias, nor does it ensure cultural neutrality. Rather, it reflects alignment with the perceptual tendencies represented in the underlying data. Transparent reporting of dataset composition, bias mitigation strategies, and their limitations is therefore essential when interpreting AI-based facial perception outputs in clinical and research contexts.

## Statistical Analysis

Pearson’s (PCC) and intraclass correlation coefficient (ICC) were calculated for each trait to evaluate the agreement between AI predictions and human ratings. The PCC (r) measures the linear relationship between two variables, producing a value between − 1 and 1. A PCC of 1 indicates a perfect positive relationship, −  indicates a perfect negative relationship, and 0 means no correlation. It is commonly used to assess the agreement between predictions and ground truth (e.g., human-rated labels) in tasks like regression or scoring. This metric is particularly valuable for evaluating how well a model captures patterns in the data rather than just individual data points.

ICC interpretations followed Cicchetti’s Guidelines [42]: ICC < 0.4 poor, 0.4 ≤ ICC < 0.6 fair, 0.6</0ICC < 0.75 moderate, ICC > 0.75 excellent.

Scatterplots of mean scores per image were used to visualize linear correlation and potential outliers.

## Results

According to Cicchetti’s guidelines, the attractiveness trait showed the following results: *r *= 0.9, ICC = 0.9 which can be interpreted as excellent correlation. The traits trustworthy, rested, happy, healthy, and sexy also reached excellent approval: trustworthy *r* = 0.84 and ICC = 0.83; rested *r* = 0.86 and ICC = 0.85; happy *r* = 0.86 and ICC = 0.85; healthy r = 0.79 and ICC 0.79; sexy *r *= 0.87; and ICC=0.86. The correlations for dominant and threatening are *r *= 0.61 and ICC = 0.58 for dominant; and *r* = 0.57 and ICC = 0.53 for threatening. These results represent a fair correlation according to Cicchetti [42], which can still be interpreted as highly significantly correlated. The naturalness prediction also shows excellent correlations: *r *= 0.87 and ICC = 0.86 (Table 6).

**Table 6:** Pearson’s (r) and intraclass correlation coefficients (ICC): AI predictions versus human raters for each trait

	Pearson’s correlation coefficient (r)	Intraclass correlation coefficient (ICC)
Attractive	0.9	0.9
Trustworthy	0.84	0.83
Rested	0.86	0.85
Happy	0.86	0.85
Healthy	0.79	0.79
Sexy	0.86	0.86
Dominant	0.61	0.58
Threatening	0.57	0.53
Naturalness	0.87	0.86

The scatter plots show that the AI predictions cover the full range of rater’s scores without significant outliers (Figs. 1, 2, 3, 4, 5, 6, 7, 8 and 9)*.*

**Fig. 1:**
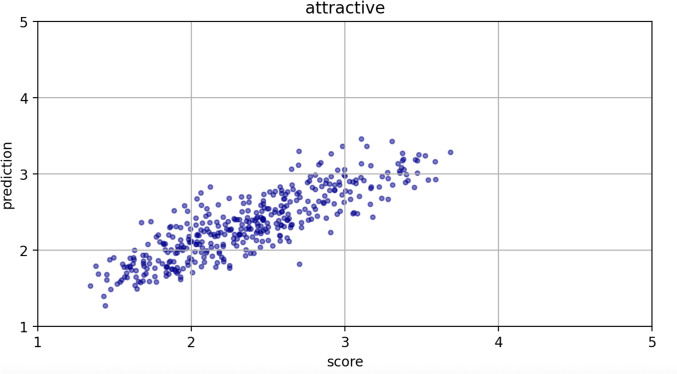
Scatterplot, comparison of the AI prediction and mean human ratings for “attractive”

**Fig. 2:**
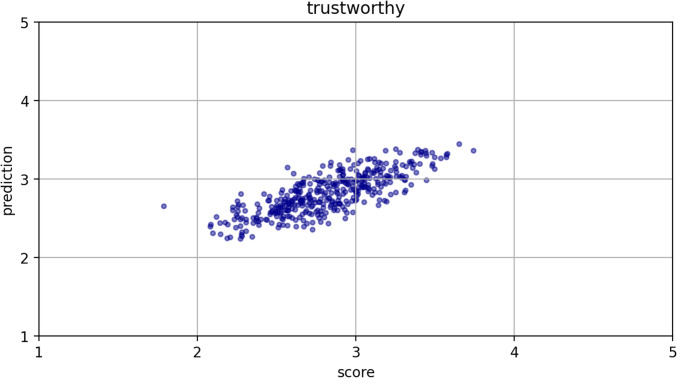
Scatterplot, comparison of the AI prediction and mean human ratings for “trustworthy”

**Fig. 3:**
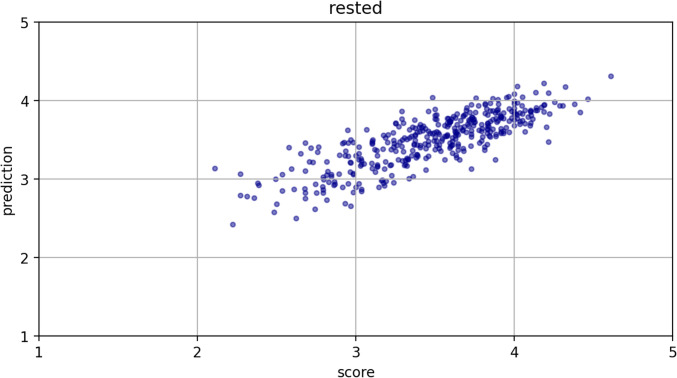
Scatterplot, comparison of the AI prediction and mean human ratings for “rested”

**Fig. 4:**
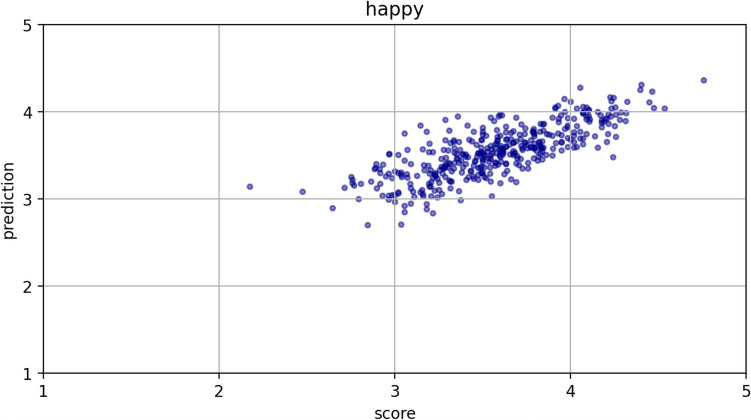
Scatterplot, comparison of the AI prediction and mean human ratings for “happy”

**Fig. 5:**
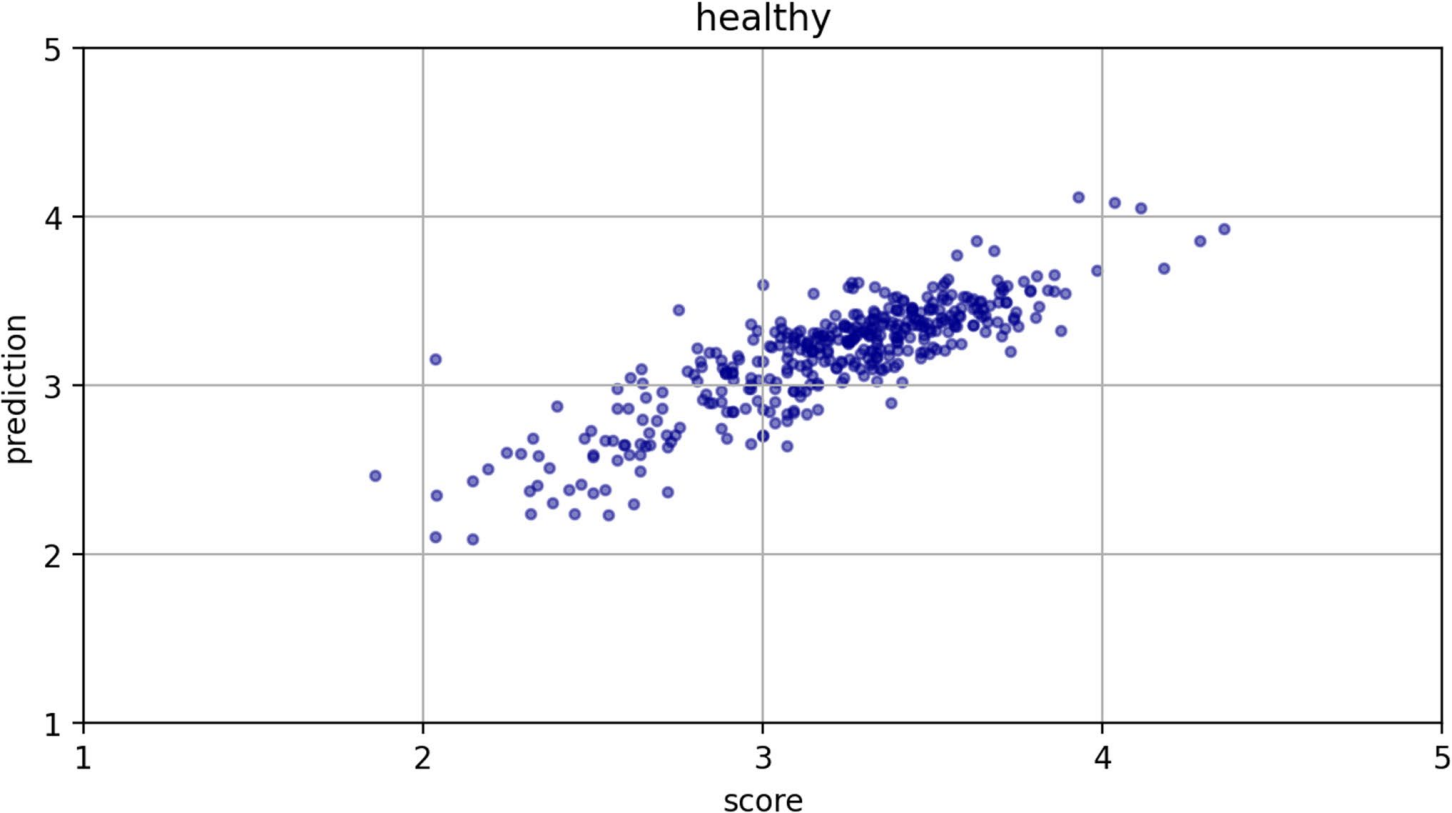
Scatterplot, comparison of the AI prediction and mean human ratings for “healthy”

**Fig. 6:**
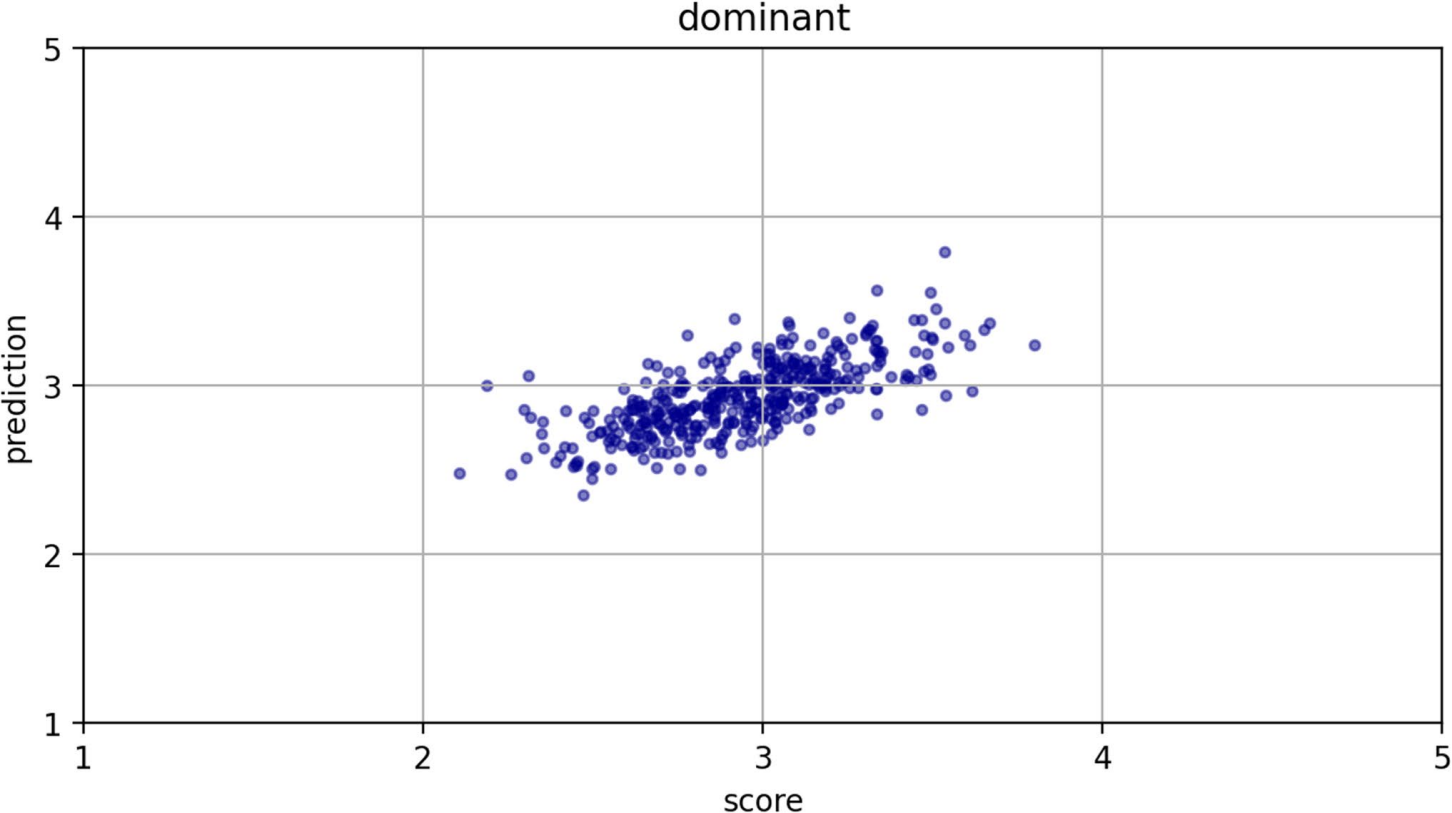
Scatterplot, comparison of the AI prediction and mean human ratings for “dominant”

**Fig. 7:**
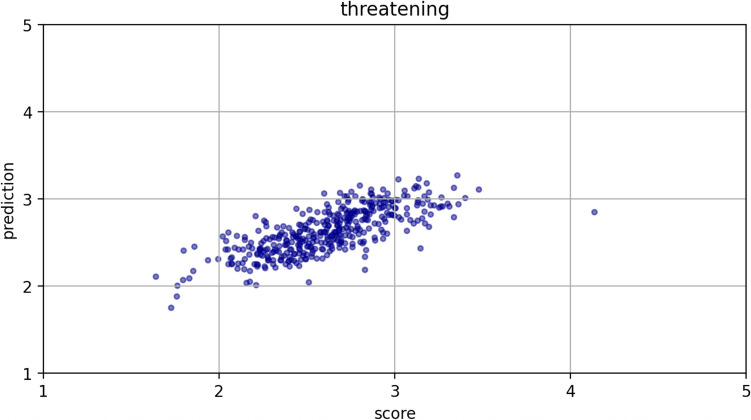
Scatterplot, comparison of the AI prediction and mean human ratings for “threatening”

**Fig. 8:**
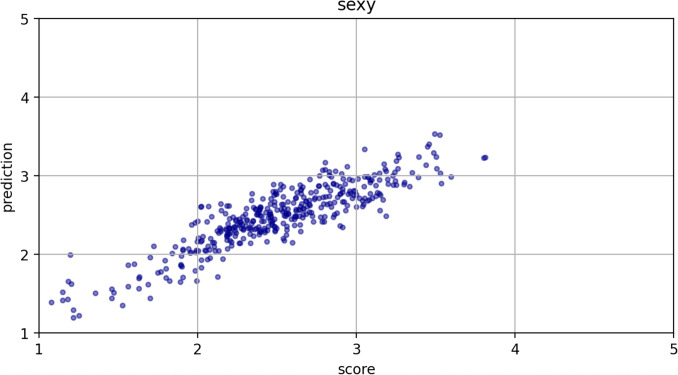
Scatterplot, comparison of the AI prediction and mean human ratings for “sexy”

**Fig. 9:**
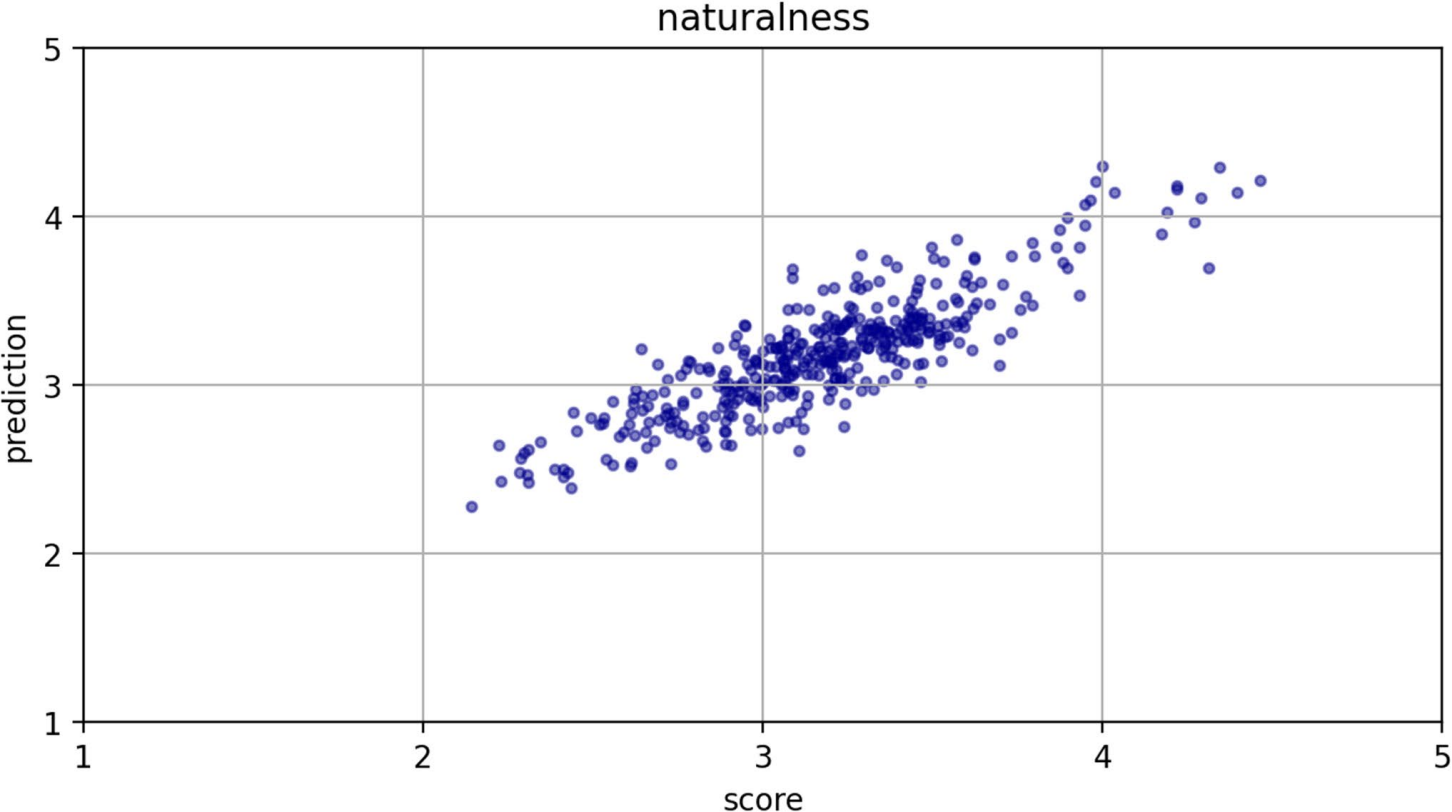
Scatterplot, comparison of the AI prediction and mean human ratings for “naturalness”

Stratified analyses by ethnicity revealed consistent correlation patterns, indicating stable model performance across demographic subgroups. Table 7 shows the PCC scores for the different ethnicities among images.

**Table 7:** Pearson’s correlation coefficients (r): AI predictions per ethnicity (images)

	Asian	Black	Latino	White
Attractive	0.861	0.834	0.877	0.922
Trustworthy	0.812	0.795	0.859	0.843
Rested	0.785	0.855	0.899	0.844
Happy	0.797	0.892	0.893	0.849
Healthy	0.799	0.700	0.790	0.821
Sexy	0.889	0.729	0.855	0.893
Dominant	0.571	0.627	0.666	0.602
Threatening	0.521	0.548	0.594	0.576
Naturalness	0.897	0.768	0.907	0.866
Average	0.773	0.738	0.809	0.796

## Discussion

### Principle Findings and Clinical Relevance

Previous literature has shown that people form social judgements based on facial cues [1, 2, 9]. While traditional psychological studies relied on controlled laboratory settings and manual annotation, the rise of deep learning enables scalable models trained on real-world images [32, 43]. This study presents the validation of a machine learning-based algorithm to quantify multiple facial first-impression traits by comparing algorithmic predictions with aggregated human consensus ratings, reinforcing the potential of artificial intelligence (AI) as an objective, scalable tool. The principle finding of the present work is that the algorithm demonstrates strong statistical agreement with human evaluators across several perception traits, particularly attractiveness, trustworthiness, healthiness, happiness, rested appearance, sexiness, and perceived naturalness. In contrast, traits such as dominance and threatening appearance show significant but fair agreement, consistent with prior evidence that these constructs are more context-dependent and less visual constraint. Importantly, the present results should be interpreted as evidence of statistical concordance with human judgements. The results however should not be seen as an ability of the algorithm to replicate the full complexity of human social perception including facial expressions. Recent literature has shown that especially beauty-standards may be dependent population specific ideals [44]. Training with diverse datasets with significant representation of all ethnicities hence becomes an important cornerstone of industry relevant AI development.

Our statistically significant results confirm the potential use of AI in assessing first impressions for specific personality traits with clear visual correlation. This aligns with previous works showing the effect of specific facial features on social perception [45]. The consistency suggests that AI models are particularly adept at recognizing features that have a biological or evolutionary basis for human perception.

The accurate modeling of subjective constructs, such as trustworthiness, healthiness, or attractiveness, using machine learning opens up new avenues for facial analysis, treatment planning, outcome evaluation, and patient communication.

**Fig. 10:**
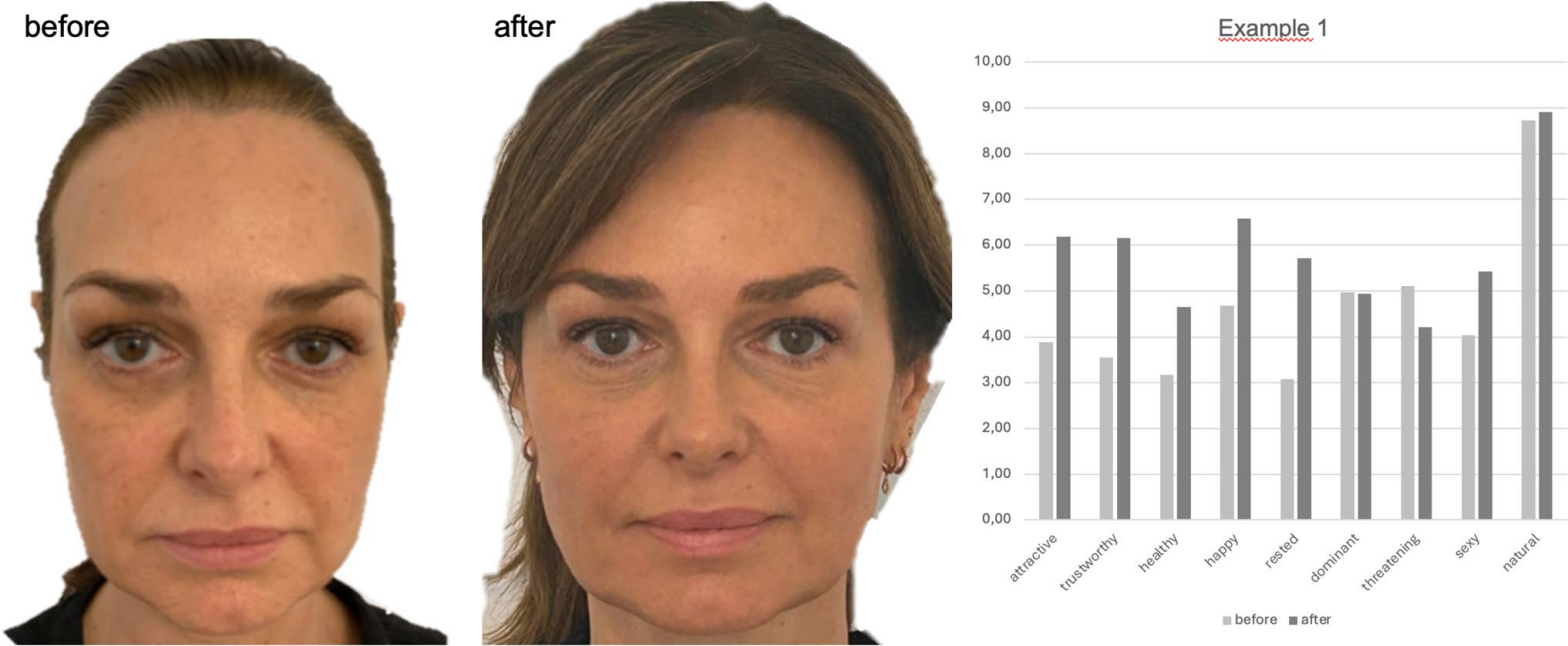
Example 1 of facial perception scores before and after aesthetic treatment (Y-axis: score 1–10, X-axis: perceptive trait, light gray: before treatment, dark gray: after treatment)

**Fig. 11:**
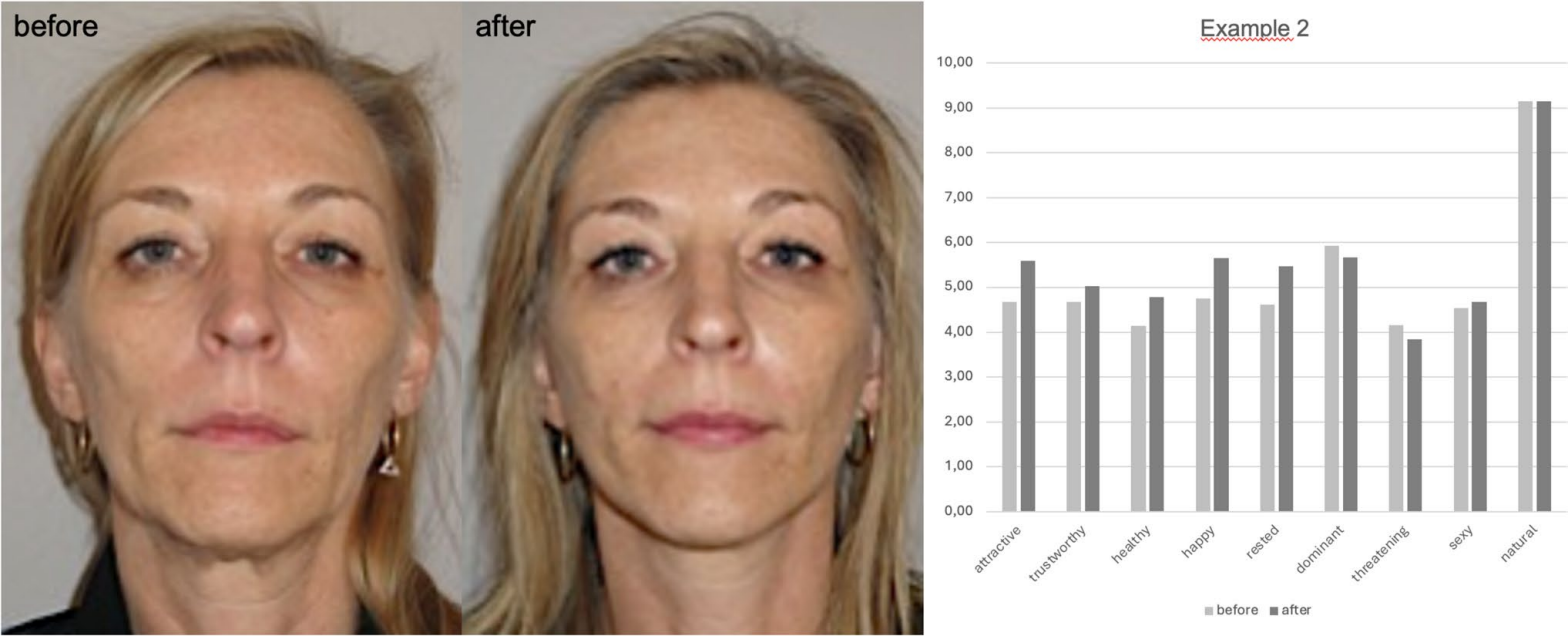
Example 2 of facial perception scores before and after aesthetic treatment (Y-axis: score 1–10, X-axis: perceptive trait, light gray: before treatment, dark gray: after treatment)

**Fig. 12:**
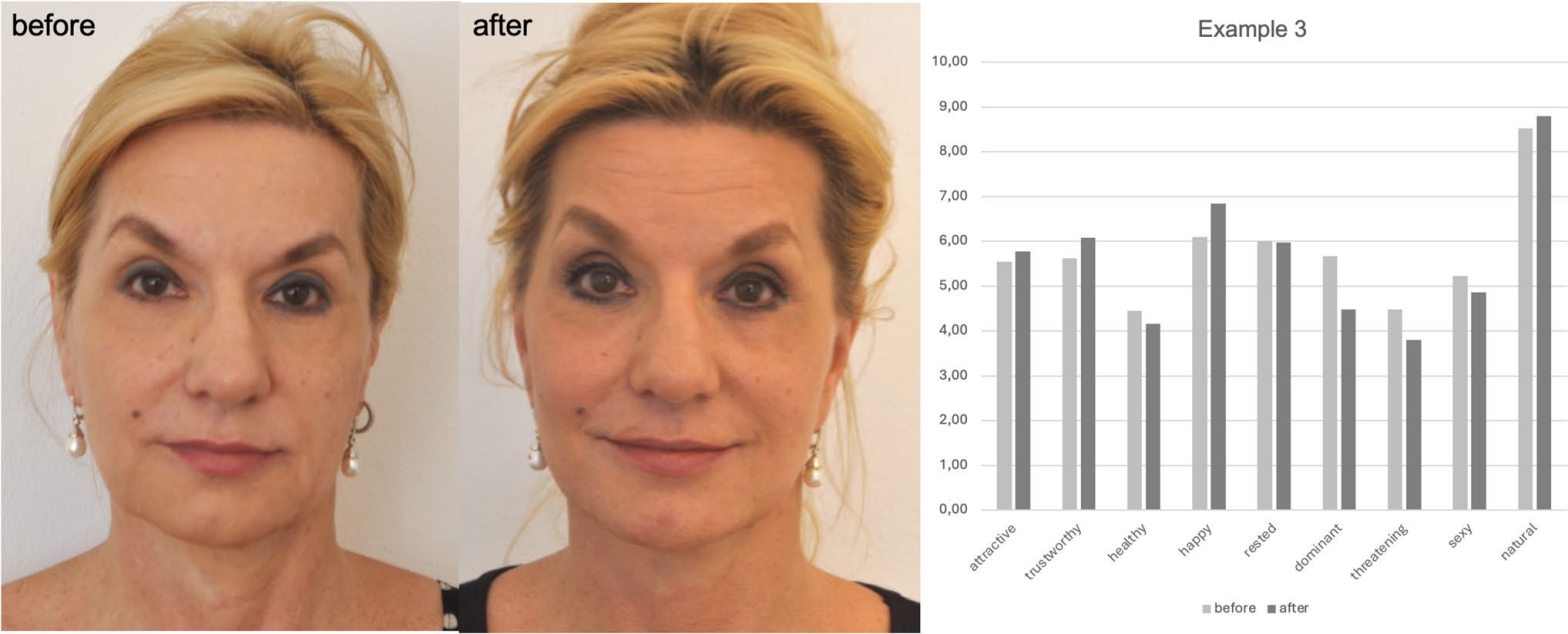
Example 3 of facial perception scores before and after aesthetic treatment (Y-axis: score 1–10, X-axis: perceptive trait, light gray: before treatment, dark gray: after treatment)

**Fig. 13:**
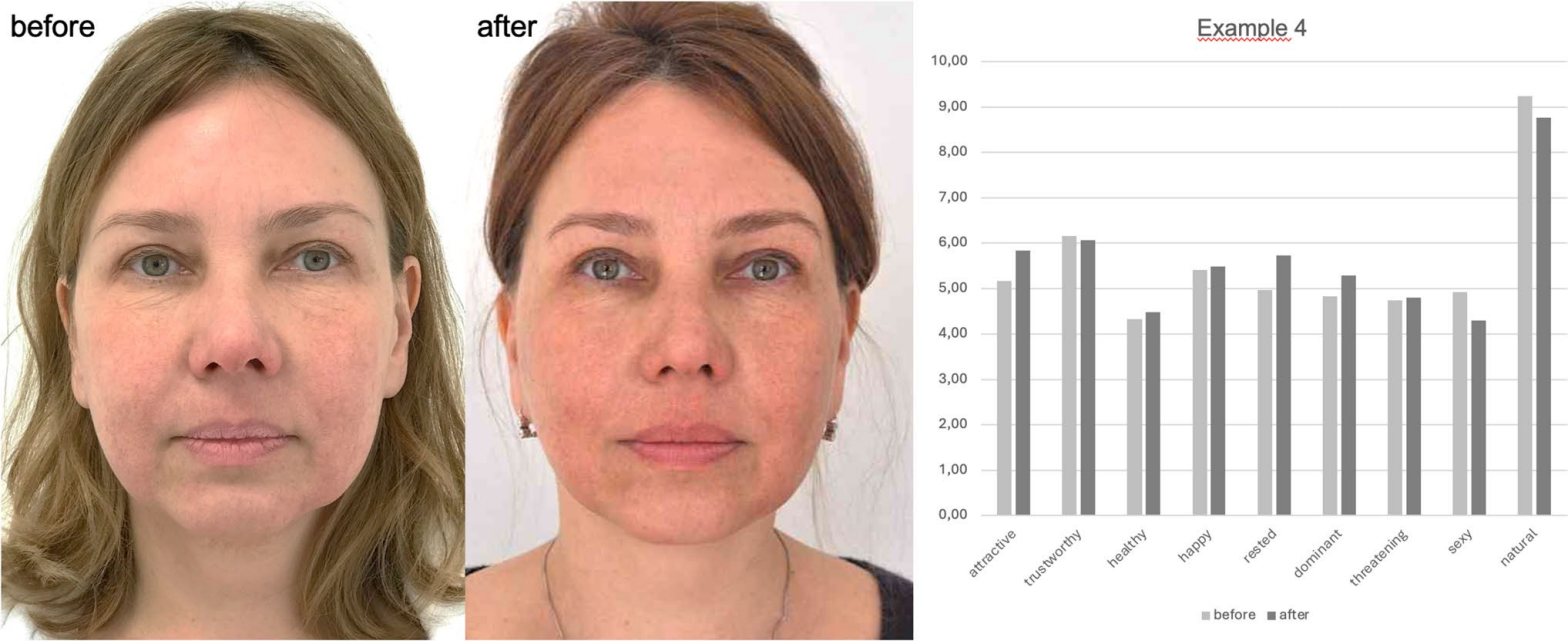
Example 4 of facial perception scores before and after aesthetic treatment (Y-axis: score 1–10, X-axis: perceptive trait, light gray: before treatment, dark gray: after treatment)

As awareness grows around the role of perceptive facial traits in shaping social and professional interactions, aesthetic medicine is undergoing a conceptual shift [18]. Traditionally, aesthetic treatments have focused on rejuvenation, but recent studies show that patients increasingly seek to influence how they are perceived in everyday life [15, 16]. With the advent of AI, particularly CNNs, clinicians and patients now have access to a validated, objective, and reproducible tool that can quantify these social perception traits [30]. This allows practitioners to align interventions with individualized goals such as enhancing how healthy a face is being perceived, rather than simply minimizing age related changes [46, 47]. To give some examples, Figures 10, 11, 12, and 13 show the changes in perceptive traits of patients before and after aesthetic treatments.

### AI Accuracy and Validation in Dermatology and Aesthetic Medicine

Due to its ability to analyze clinical and aesthetic features with high accuracy, AI has gained significant traction in dermatology and aesthetic medicine.

AI-based diagnostic systems have achieved performance on par with dermatologists. For instance, a deep learning system developed by Liu et al. for tele-dermatology achieved a top-1 diagnostic accuracy of 66%, matching dermatologists (63%), and outperforming primary care physicians and nurse practitioners (44 and 40%, respectively) [48]. Systematic reviews by Gomes et al. and Kania et al. confirm that deep neural networks consistently demonstrate high accuracy and sensitivity in classifying skin conditions and assessing treatment outcomes, including wrinkle severity, skin hydration, and sebum content [46, 49]. Recent systematic reviews of AI in surgery have consistently highlighted substantial heterogeneity in validation quality, limited external validation, and lack of standardized frameworks for assessing clinical readiness [36]. In their comprehensive review of more than 100 AI studies, Kenig and colleagues demonstrated that fewer than half of published models met high-evidence validation criteria and that publicly available datasets were rare.

Within facial aesthetic surgery, AI applications have primarily focused on age estimation, attractiveness prediction, surgical outcome simulation, or detection of prior aesthetic intervention [50]. While these studies illustrate technical feasibility, most emphasize algorithmic performance rather than structured validation against human consensus ratings or systematic assessment of bias. The present study directly addresses these gaps by providing agreement-based metrics and subgroup analyses, thereby aligning with recent calls for increased methodological rigor and transparency in AI research [36, 38].

## Limitations

Despite the transformative potential, the integration of AI in aesthetic medicine faces several limitations. First: One major concern is algorithmic bias, which arises when training datasets lack diversity across ethnicity and age which can lead to inaccurate or non-inclusive results [51, 52]. Multiple reviews have shown that AI models trained on non-representative datasets risk perpetuating demographic bias [50, 53]. This limitation was addressed in our study by including a relevant distribution of ethnicity, gender, and age to our population, in facial images and raters respectively. Subgroup analyses demonstrated consistent correlation patterns across major ethnic groups. Where we identified significant imbalances or biases, label adjustments were performed during data preprocessing to counteract disparities and create more equitable and accurate AI model outcomes. However, these measures cannot eliminate the possibility of latent bias embedded in human ratings themselves. As emphasized in recent ethical frameworks, bias mitigation is an ongoing process that requires transparent reporting [53, 54]. Second: Conceptual overlap among perceptual traits limits strict separation of constructs, which might be of minor clinical relevance. Several assessed traits—such as attractiveness, trustworthiness, healthiness, and happiness—are inherently subjective and strongly interrelated. Psychological research has shown that positive facial evaluations in one domain often generalize to others, a phenomenon commonly referred to as the “halo effect” [2]. This interdependence may inflate correlations and introduce construct-related bias. In addition, the trait labeled “naturalness” warrants clarification. In this study, naturalness reflects perceived likelihood of prior aesthetic intervention based solely on visual appearance. It does not represent an objective assessment of surgical history or aesthetic quality. Distinguishing social perception from aesthetic preference and personality attribution is essential to avoid misinterpretation or overextension of algorithmic outputs. Third: there is a limited standardization in AI validation protocols within aesthetic contexts. As Nogueira et al. [47] and Spoer et al. [55] note, many AI models remain in preclinical phases with few externally validated or regulated for real-world use. This is why it was of great importance to validate our AI model for measurement of facial perceptive traits. These limitations preclude any claim that AI can detect subtle aspects of human appearance with perfect or universal accuracy; however, statistical significance outperforms conventional methods.

## Ethical and Regulatory Considerations

Recent ethical reviews and guidelines in aesthetic medicine have identified recurring concerns, including data privacy, algorithmic opacity, unclear liability, and insufficient regulatory oversight [38, 53, 54], particularly in AI models trained on sensitive facial images [56], which makes an informed consent mandatory. AI-driven simulations may exaggerate results, mislead patients, or perpetuate narrow aesthetic ideals [57]. Regulatory oversight is necessary to enforce standards for inclusivity and transparency in model development [30]. Therefore, the main reason to publish our training and validation data was to show the reliability of our system and underscore that we adequately addressed bias issues.

Crucially, the present study does not propose autonomous AI-driven decision making. By restricting the algorithm’s role to measurement, validating it against human consensus, and explicitly framing it as a supportive tool, this work addresses several ethical requirements emphasized in the literature.

## Future Research

Future research should focus on external and longitudinal validation, integration of three-dimensional or dynamic facial data, and correlation of AI-derived perception outcomes and clinically meaningful endpoints. Expanded cross-cultural validation will be essential to enhance interpretability, generalizability, and ethical robustness, as emphasized in recent surgical AI frameworks^36^, ^53^. Research should also explore how AI-derived perception scores can inform clinical decisions, select certain techniques, prevent disadvantageous outcomes, improve communication between practitioners and patients, and enhance treatment planning.

## Conclusion

This study provides a rigorous validation of an AI-based approach to quantify first-impression traits, demonstrating meaningful agreement with human judgements while explicitly acknowledging conceptual, methodological, and ethical constraints. When applied transparently, the close alignment between AI predictions and human consensus underscores the model´s potential utility as a valuable clinical tool for facial assessment, decision support as well as outcome measurement in terms of education and research.
